# Spontaneous Healing of Iatrogenic Complete Ureteric Transection Injury

**DOI:** 10.7759/cureus.19440

**Published:** 2021-11-10

**Authors:** Hani Sayedin, Mohammad Al-Machhour

**Affiliations:** 1 Urology, Warrington and Halton Teaching Hospitals NHS foundation Trust, Warrington, GBR; 2 Urology, Warrington and Halton Teaching Hospitals NHS Foundation Trust, Warrington, GBR

**Keywords:** reconstrictive urology, genito-urinary pathology, post hysterotomy complication, iatrogenic ureteric injury, ureteric trauma

## Abstract

Iatrogenic ureteric injury is the most common cause of ureteric injury. It is usually caused by either gynecological or urological surgical procedures. Iatrogenic ureteric injury repair depends mainly on the time of diagnosis. We represent here a case of iatrogenic complete transection ureteric injury resulted from laparoscopic bilateral salpingo-oophorectomy. The patient had a history of abdominal hysterectomy causing adhesions that resulted in challenging surgery. One week later, the patient presented to the emergency department with abdominal pain, and contrast CT showed left hydronephrosis with extravasation of the contrast at the left renal pelvis. The patient was treated initially with left nephrostomy and an antegrade nephrostogram confirmed the diagnosis of complete transection ureteric injury. Surprisingly, left retrograde study, which was done 11 weeks after the operative injury, showed healing of the ureteric injury with a small annular stricture. The stricture was dilated and a stent was inserted. We concluded that conservative waiting and delayed ureteric repair might be advised in similar injuries allowing time for resolution of the postoperative inflammatory reaction and spontaneous healing.

## Introduction

The ureter might be protected by its small size and the surrounding structures including viscera, pelvic bony wall, and back muscles. Therefore, iatrogenic ureteric injury is the most common cause of ureteric injury and is considered a major surgical complication [1]. Fifty percent to 80% of iatrogenic ureteric trauma occurred during gynecological surgery with about 1.3 iatrogenic ureteric injuries in every 1000 hysterectomies [2]. Ideally, iatrogenic ureteric injury should be diagnosed intraoperatively and immediate repair should be performed. However, about 70% of injuries are diagnosed postoperatively [3]. Urinary drainage is the role and is usually performed with either ureteric stent or nephrostomy, depending on the clinical situation. Therefore, a low threshold of ureteric injury suspicion should be considered. A contrast study is needed to confirm the diagnosis, either contrast CT or retrograde pyelography. Contrast extravasation is the hallmark sign for ureteric injury, but also mild ureteric dilatation and urinoma might be the only signs [4].

Many reconstructive techniques are utilized for ureteric injury repair depend on the site of the injury. In distal ureteric injuries, ureteroneocystostomy is usually performed, either by refluxing or non-refluxing techniques. Vesico-psoas hitch could be performed to reduce the tension of the neo-anastomosis [5]. In mid and upper ureteric injuries, variable reconstructive techniques could be performed depending on the defect size, the level of injury, and surgical preference, including ureteroureterostomy, Boari flap, transureteroureterostomy, renal autotransplantation, and ureteral substitution with ileal segment [6]. However, most of these reconstructive surgeries are complex procedures with relatively high index postoperative complications including leakage, stricture, and deterioration of the renal unit function. Therefore, the conservative management, using nephrostomy tube and/or ureteric stent either antegrade or retrograde, should be tried initially before stepping forward to reconstructive surgery [7]. Our case represents the efficacy of conservative management in dealing with iatrogenic injury. In addition, to the maximum of our knowledge, there is no specific recommendation for the time window needed before the deferred reconstructive surgery.

## Case presentation

**Figure 1:**
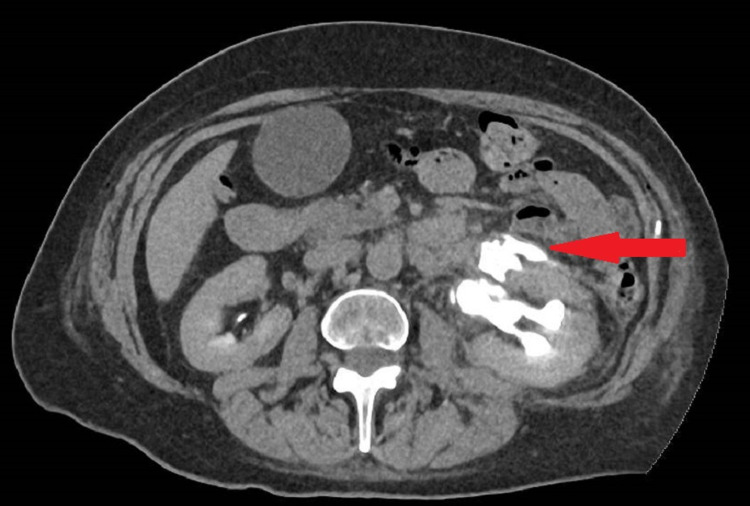
Axial contrast CT scan showing extravasation of the contrast at the left renal pelvis one week post traumatic injury (red arrow)

**Figure 2:**
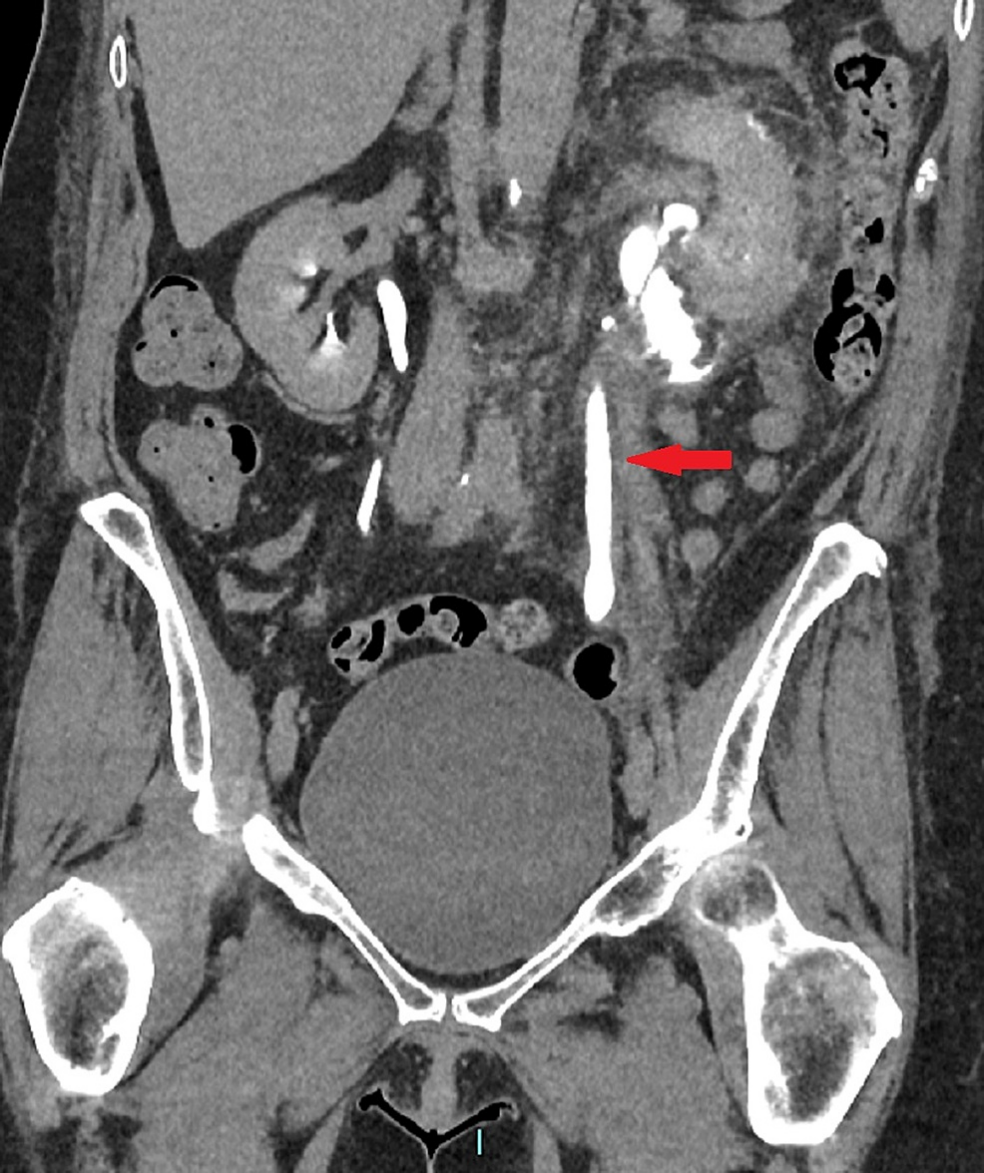
Coronal contrast CT showing mild dilatation of the left ureter in comparison with the other side one week post traumatic injury (red arrow)

A 56-year-old woman underwent laparoscopic bilateral salpingo-oophorectomy by the gynecological team. The patient has a history of total abdominal hysterectomy 20 years prior for endometriosis. Therefore, adhesions were obscuring the anatomy of the left ureter during her later surgery. In the early postoperative days, there was mild pain at the left iliac fossa and was managed with paracetamol and oral morphine. In the early period, there was no flank pain and no costovertebral angle tenderness. One week later, the patient presented to the emergency department with severe left iliac fossa pain associated with nausea and vomiting. Her investigations were within normal range, stable renal function, and normal inflammatory markers. Contrast CT showed extravasation of the contrast at the level of the left renal pelvis (Figure 1) and ureteric dilatation down to the pelvic ureter (Figure 2).

**Figure 3:**
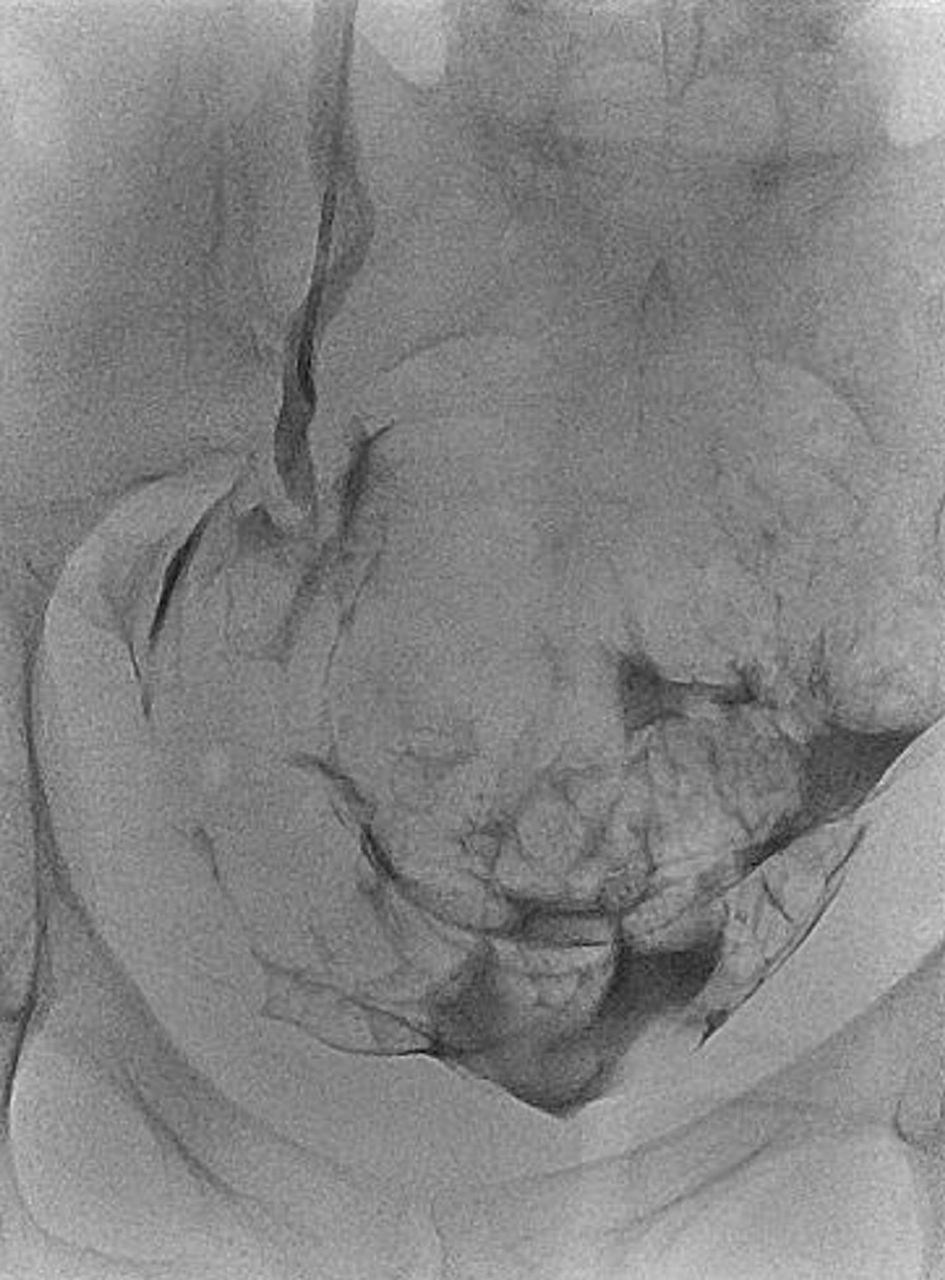
Fluoroscopy image - left antegrade study showing extravasation of the contrast into the peritoneal cavity three weeks post injury

**Figure 4:**
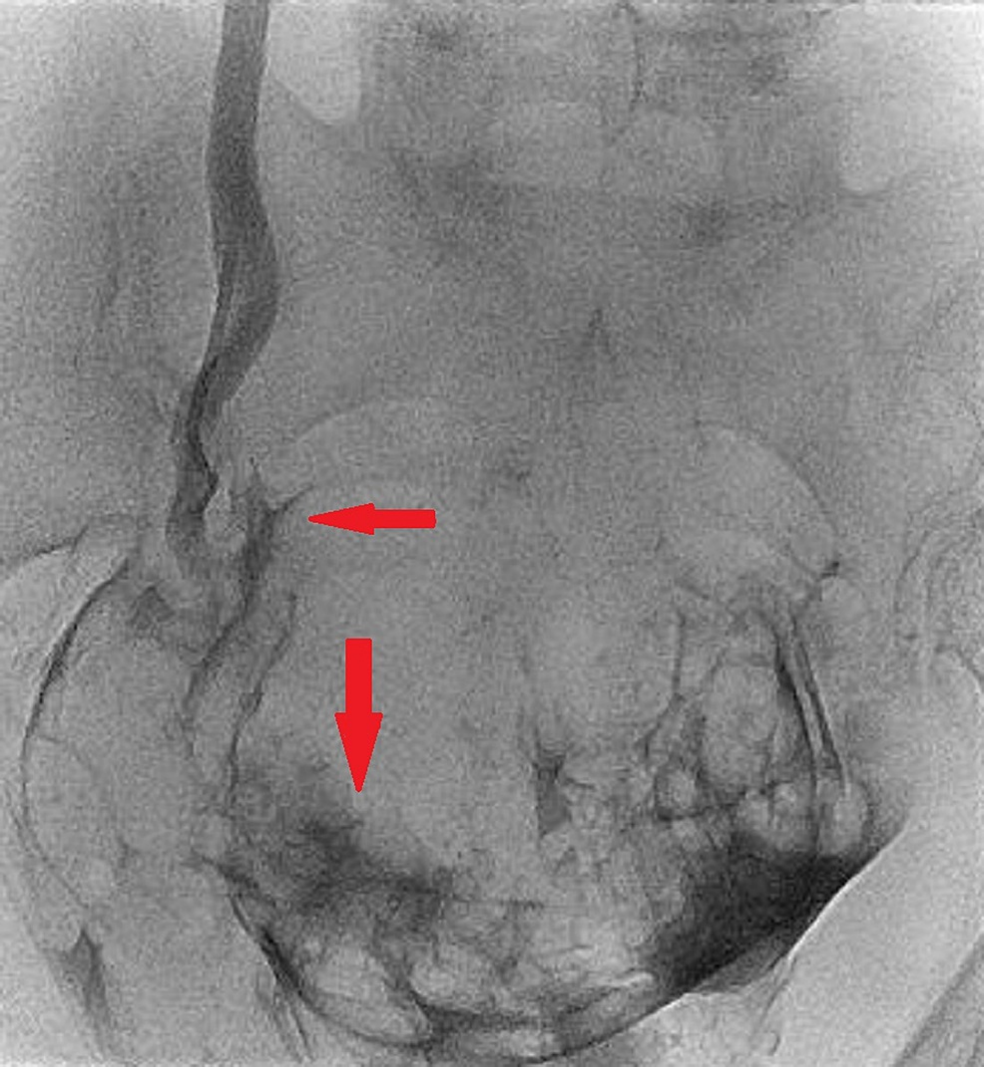
Fluoroscopy image - left antegrade study showing contrast extravasation at the injury site three weeks post injury (red arrows)

Considering the recent pelvic surgery, immediate diagnosis of left ureteric iatrogenic injury was concluded, and the patient was managed initially with urgent CT-guided nephrostomy insertion. The patient was in pain and an adequate nephrostogram was not performed during nephrostomy insertion. Therefore, three weeks posttraumatic injury, the patient underwent a proper antegrade nephrostogram. The contrast extravasated into the peritoneal cavity and there was no contrast passing into the distal left ureter (Figures 3, 4). Therefore, a diagnosis of complete transection of ureteric injury was suspected. The nephrostomy was left in situ and the patient was planned for reconstructive surgery. 

**Figure 5:**
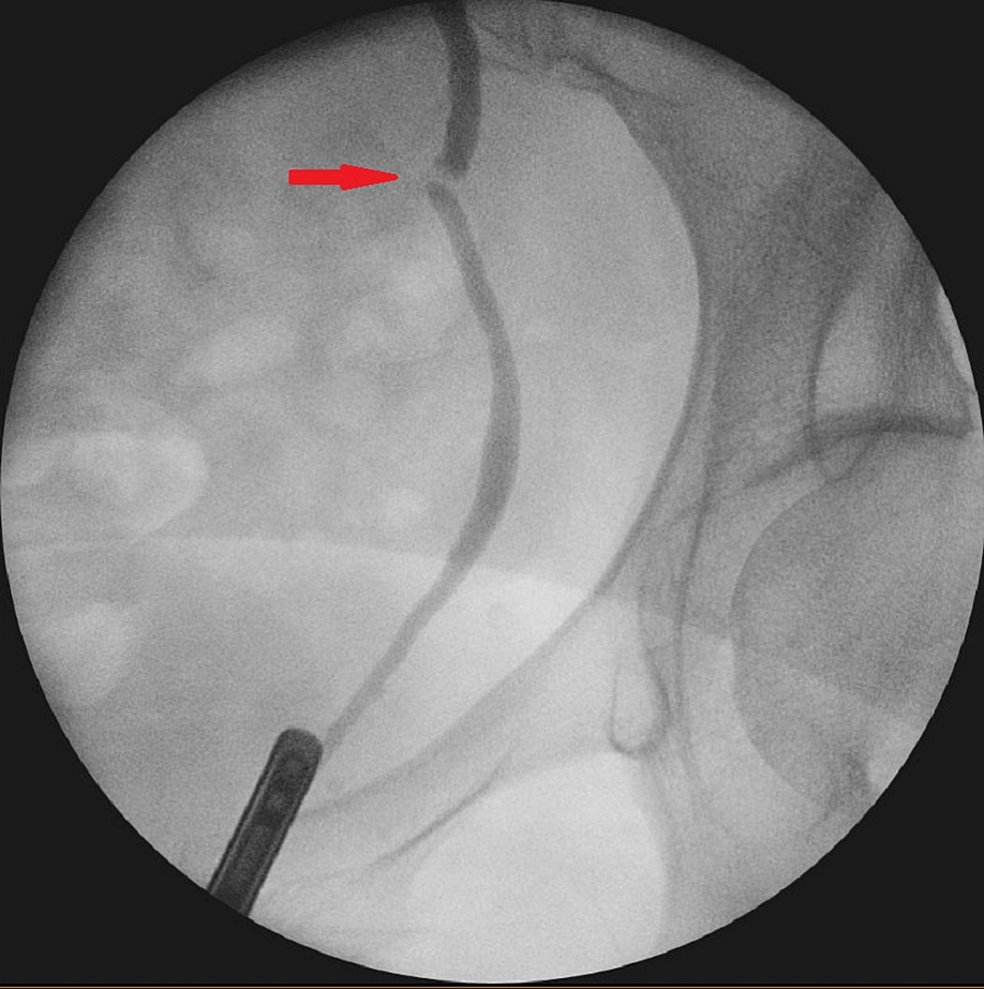
Left retrograde study showing a small annular stricture at the site of the injury, and the contrast ascend in the left ureter with no extravasation 11 weeks post traumatic injury (red arrow)

**Figure 6:**
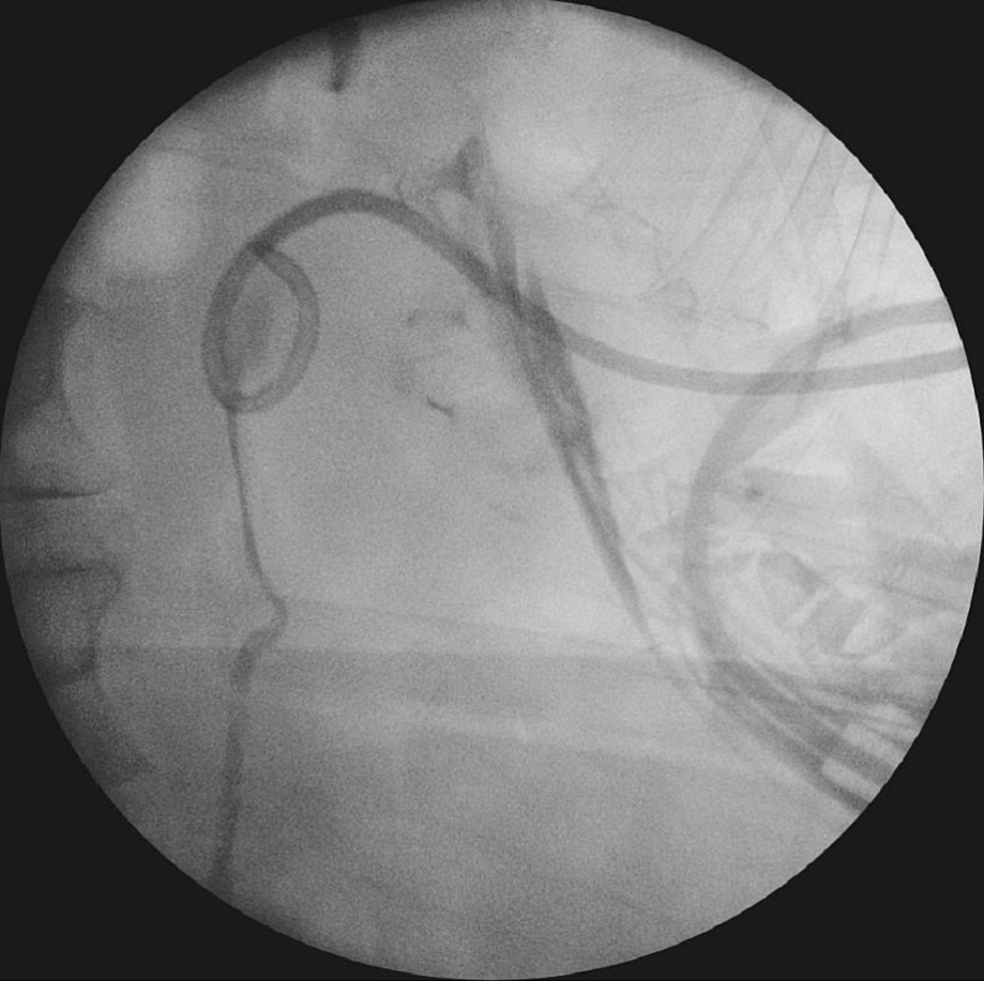
Left retrograde study showing the contrast ascended to the left kidney 11 weeks post traumatic injury

Eleven weeks posttraumatic injury, the patient was admitted for her planned surgery. Prior to the surgery, left retrograde study was performed and surprisingly the contrast went up to the left kidney with only annular stricture at the level of the injury (Figures 5, 6). Therefore, a decision was taken for ureteroscopic dilatation of the stricture. The ureteroscopy showed a small annular stricture, less than 0.5 cm, that was passed easily over the safety guidewire and a ureteric stent, size 8 French, was inserted successfully.

## Discussion

The clinical presentation and the contrast CT findings of the patient suggested obstruction at the level of the pelvic ureter either by ligature or cauterization injury (Figures 1, 2). In addition, the antegrade study appearance showed total contrast extravasation suggesting complete transection injury (Figures 3, 4). However, the retrograde study showed that the ureter might spontaneously heal by stricture after urinary diversion by nephrostomy tube (Figures 5, 6). This clinical scenario would confirm the importance of conservative “waiting” in iatrogenic ureteric injury. Iatrogenic injury, as discussed before, is usually discovered later [3]. Punekar et al. reviewed ureteric stricture dilatation in 16 patients and they reported a success rate of 69% [8]. They concluded that the success rate will be affected by three factors, such as age, length, and cause of the stricture. The age of stricture is favorable to be less than one year, the length is favorable to be less than 2 cm, and the cause to be postsurgical is more favorable than posttuberculous strictures. In addition, they reported the success rate in postsurgical stricture is nearly 100% with the advantage of being minimally invasive management. The three criteria were applied to our case. Therefore, a decision of minimally invasive ureteric dilatation was decided rather than complex reconstructive injury. The limitation of this study is that we do not have the long-term result of the stricture dilatation. 

The real extent of the iatrogenic injury and its related defect would be difficult to be assessed exactly and it will be estimated only through contrast studies. However, bridging epithelial or even adventitial ureteric tissue between the edges of the defect might help the healing process. Therefore, “conservative waiting” should be always adopted when the clinical condition allows, hoping for healing and epithelization over the area of defect.

## Conclusions

Iatrogenic ureteric injury is commonly diagnosed lately, and the real extent of the ureteric injury might be difficult to be assessed exactly. Reasonable “conservative waiting” for about three months might be recommended when possible provided that the affected kidney is adequately drained with nephrostomy tube. If conservative waiting would be applied, it might allow for the postoperative inflammatory reactions to subside and spontaneous healing might occur, sparing the patient complexed reconstructive surgeries.
